# The Utility of Home-Practice in Mindfulness-Based Group Interventions: A Systematic Review

**DOI:** 10.1007/s12671-017-0813-z

**Published:** 2017-09-23

**Authors:** Annette Lloyd, Ross White, Catrin Eames, Rebecca Crane

**Affiliations:** 1 Institute of Health and Well-Being, University of Glasgow, Glasgow, UK; 2 Psychology Services, NHS Greater Glasgow and Clyde, Glasgow, UK; 3 School of Psychology, University of Liverpool, Liverpool, UK; 4 Centre for Mindfulness Research and Practice, School of Psychology, Bangor University, Brigantia Building, Gwynedd, UK

**Keywords:** Mindfulness-based stress reduction, Mindfulness-based cognitive therapy, Mindfulness-based interventions, Home-practice

## Abstract

A growing body of research supports the efficacy of mindfulness-based interventions (MBIs). MBIs consider home-practice as essential to increasing the therapeutic effects of the treatment. To date however, the synthesis of the research conducted on the role of home-practice in controlled MBI studies has been a neglected area. This review aimed to conduct a narrative synthesis of published controlled studies, evaluating mindfulness-based group interventions, which have specifically measured home-practice. Empirical research literature published until June 2016 was searched using five databases. The search strategy focused on mindfulness-based stress reduction (MBSR), mindfulness-based cognitive therapy (MBCT), and home-practice. Included studies met the following criteria: controlled trials, participants 18 years and above, evaluations of MBSR or MBCT, utilised standardised quantitative outcome measures and monitored home-practice using a self-reported measure. Fourteen studies met the criteria and were included in the review. Across all studies, there was heterogeneity in the guidance and resources provided to participants and the approaches used for monitoring home-practice. In addition, the guidance on the length of home-practice was variable across studies, which indicates that research studies and teachers are not adhering to the published protocols. Finally, only seven studies examined the relationship between home-practice and clinical outcomes, of which four found that home-practice predicted improvements on clinical outcome measures. Future research should adopt a standardised approach for monitoring home-practice across MBIs. Additionally, studies should assess whether the amount of home-practice recommended to participants is in line with MBSR/MBCT manualised protocols. Finally, research should utilise experimental methodologies to explicitly explore the relationship between home-practice and clinical outcomes.

## Introduction

There is no clear consensus regarding the definition of ‘mindfulness’ (Anālayo 2016); however, a widely cited description suggests that mindfulness involves ‘paying attention in a particular way: on purpose, in the present moment, and non-judgmentally’ (Kabat-Zinn 1994, p. 4). Mindfulness is the core attentional stance underlying all types of Buddhist meditative practice. In these traditions, the formal practice of mindfulness is embedded within a larger conceptual, spiritual and practice-based ethical framework directed towards non-harming (Kabat-Zinn 2003). This includes a skilful understanding of how unexamined behaviours and an ‘untrained mind’ can contribute to human suffering and how formal meditative practices can calm and clarify the mind, refine attention and action and open the heart to transform this suffering. Mindfulness has been developed within the Buddhist tradition over the last 2500 years, but it is over the last 40 years that these Buddhist traditions have taken root in mainstream contexts (Kabat-Zinn 2003).

There has been growing interest in the effectiveness of mindfulness-based interventions (MBIs) in clinical settings. An increasing body of research supports the efficacy of various forms of MBIs, including Mindfulness-Based Stress Reduction (MBSR; Kabat-Zinn 2013) and Mindfulness-Based Cognitive Therapy (MBCT; Segal et al. 2013), for a wide range of psychological, medical and psychosomatic conditions (Grossman et al. 2004; Keng et al. 2011). MBSR was developed by Jon Kabat-Zinn and is a highly structured skill-based educational programme that combines training in mindfulness meditation with contemporary approaches to stress (Kabat-Zinn 2013). MBCT was developed by Segal et al. (2013) and is a manualised 8-week group intervention of similar structure that integrates Kabat-Zinn’s MBSR programme, with cognitive therapy theory and exercises (see Santorelli et al. (2017) for MBSR curriculum guide and Segal et al. (2013) for MBCT curriculum guide).

As the amount of research evidence investigating the efficacy of MBIs increases, interest in identifying the mechanisms by which they lead to symptom improvement has also grown (Carmody and Baer 2008; Del Re et al. 2013; Hawley et al. 2014; Nyklíček and Kuijpers 2008). One aspect of MBIs hypothesised to be important for positive outcomes is *home-practice*. Home-practice in this context is a set of mindfulness practices that are assigned to participants by MBI teachers to be completed between sessions and continued after the intervention has ended. Both MBSR and MBCT emphasise the importance of daily mindfulness practice throughout the programme that is either formally or informally structured. Formal practices involve providing participants with guidance on the nature and content of a meditation practice for a specific length of time. These practices include exercises such as body scan, sitting meditation and mindful movement. Throughout the intervention, participants are also encouraged to generalise through informal practice by bringing mindful awareness to routine everyday experiences; these practices are less structured and therefore sometimes are not given a set length of time (Hawley et al. 2014). Published MBI curriculum guides outline the following home-practice: MBSR, 45 min per day of formal mindfulness practice and 5–15 min of informal practice, 6 days per week during the intervention (Santorelli et al. 2017); MBCT, 45 min of formal mindfulness practice 6 days per week and informal mindfulness practice for the duration of the intervention (Segal et al. 2013).

MBIs consider the combination of between-session and post-programme practice (henceforth referred to as ‘home-practice’) as one of the most essential components to increasing the therapeutic effects of the treatment (Vettese et al. 2009). This is mirrored in other therapeutic interventions with home-practice assignments being highlighted as a critical and key component of efficacious psychotherapy (Kazantzis et al. 2004). Regular home-practice of taught strategies has been posited to affect a number of purported cognitive behavioural mediators of psychopathology, including rumination, stress reactivity, self-criticism and experiential avoidance—factors identified as underlying a number of disorders such as depression, anxiety and addiction (Hawley et al. 2014; Vettese et al. 2009).

Although home-practice is assumed to be an important contributor to the clinical changes found in MBIs, this relationship remains somewhat unclear, and there has been little by way of a systematic review of evidence relating to this in the literature published to date. Baer (2003) conducted an empirical review of 21 mindfulness intervention studies, of which only three studies reported total home-practice during the intervention and four studies reported home-practice at follow-up. Two studies investigated the relationship between home-practice and clinical change as assessed by outcome measures, with mixed results (Astin 1997; Kristeller and Hallett 1999). Vettese et al. (2009) conducted one of the first reviews of home-practice in MBCT and MBSR and its relationship to mindfulness programme outcomes. This review identified 24 controlled and non-controlled studies that evaluated the associations between home-practice and measures of clinical functioning. Eight of the studies provided support for a positive relationship between amount of home-practice and improvement in clinical outcome measures. An additional five studies reported mixed findings, identifying support for this relationship on some measures, as well as an absence on at least one outcome measure. The remaining 11 studies did not find the expected relationship between home-practice and clinical outcomes. Parsons et al. (2017) conducted the most recent review in this area and found that across 43 MBI studies, participants completed about 60% of assigned formal mindfulness home-practice during the intervention period. There are however some important issues that these existing reviews did not address. Vettese et al. (2009) did not examine the guidance given to participants on home-practice or whether studies met the recommendations outlined by the MBIs. In addition, it only included studies that conducted analyses linking home-practice to clinical outcomes. Similarly, Parsons et al. (2017) opted to have a broad focus on evaluating studies that used a range of designs with varying degrees of methodological rigour. They investigated whether participants completed their assigned formal practice and the association between formal practice and treatment outcomes. Across 28 studies, they reported a small but significant association between participants’ self-reported formal home-practice and intervention outcomes across clinical and nonclinical populations. As with the Vettese et al. (2009) review, Parsons et al. (2017) also did not explore in detail the formal and informal home-practice guidance that was provided to participants, specifically in controlled research trials.

The findings in these reviews go some way to addressing uncertainty regarding whether home-practice influences outcome measures used to evaluate mindfulness interventions (Hawley et al. 2014). There continues to be a disparity between what is recommended clinically and what is known empirically regarding the effects of home-practice. Given the emphasis placed on home-practice and the considerable time commitment required of participants to complete practice exercises, it is imperative that understanding is improved about the potential associations between home-practice and clinical benefits. It also raises key questions regarding: the way in which mindfulness home-practice is measured across studies; what guidance is given to participants regarding the completion of home-practice; and whether the reported home-practice in studies meet the recommendations set out by MBSR and MBCT protocols. Answering these questions will be important for developing our understanding of the role of home-practice in MBIs.

The aim of this systematic review was to conduct a narrative synthesis and appraisal of methodological quality of controlled trials that have evaluated mindfulness-based (MBSR and MBCT) group interventions and have measured home-practice. Specifically, the review aimed to investigate the following questions: How did the included studies monitor home-practice? What guidance and resources were participants in the included studies given to complete home-practice? Did the study protocols of the included studies meet the requirements of guidelines for home-practice that have been stipulated for MBSR (Santorelli et al. 2017) and MBCT (Segal et al. 2013)? Finally, were higher levels of home-practice associated with better participant clinical outcomes in the included studies?

## Method

### Protocol

This review was conducted in accordance with the PRISMA statement: http://www.prisma-statement.org (Moher et al. 2009).

### Search Strategy

First, a search of the Cochrane Database of Systematic Reviews was completed to identify existing systematic reviews, meta-analyses and literature reviews. Thereafter, five databases (Web of Science Core Collection, EBSCO Psychinfo, Ovid Medline, EBSCO CINAHL and Cochrane Library) were searched from inception to September 2017 for empirical articles. A number of search terms were initially developed to decipher what combination would incorporate the widest span of research. The final search criteria utilised was *mindfulness-based stress reduction* or *MBSR* or *mindfulness-based cognitive therapy* or *MBCT* or *mindfulness* combined with *home-practice* or *homework* or *between-session practice*. Reference lists of all potentially relevant articles and other reviews were assessed to identify any studies that may have been missed. Finally, the ‘Mindfulnet’ website (www.mindfulnet.org) and the journal *Mindfulness* were reviewed for relevant studies. All titles and abstracts were reviewed, and if studies met the eligibility criteria, they were read in full independently by the first author (AL). Any ambiguities regarding whether a study met the inclusion criteria were discussed between the first (AL) and second (RW) authors to resolve any uncertainty.

### Eligibility Criteria

Studies included in the review were controlled research trials, available in English, and published in peer-reviewed journals. In addition, included studies implemented a MBSR or MBCT group intervention. Modified MBSR interventions with reduced treatment time (7 weekly, 1½–2 hr sessions) for patients with cancer were also included. Studies that included interventions for individuals with cognitive impairment or a learning disability were excluded. Studies needed to recruit participants aged 18 years and above and have collected primary data using standardised quantitative outcome and/or process measures for inclusion. Finally, studies that measured home-practice daily or weekly throughout the duration of the group intervention and/or at follow-up were included. Home-practice was operationalised as: participants practicing a set of tasks assigned to them by their group teacher to be completed outside of the group session. ‘Measurement’ of home-practice was defined as including either or both of the following: participants were asked to log the frequency of their home-practice using a self-report measure such as a log/dairy/questionnaire/calendar or home-practice was tracked objectively through electronic means (e.g. a mobile phone app). This review was interested to focus on home-practice as reported in research papers, to examine the means and variability of the reporting of this information in academic papers to date.

### Search Outcome

**Fig. 1:**
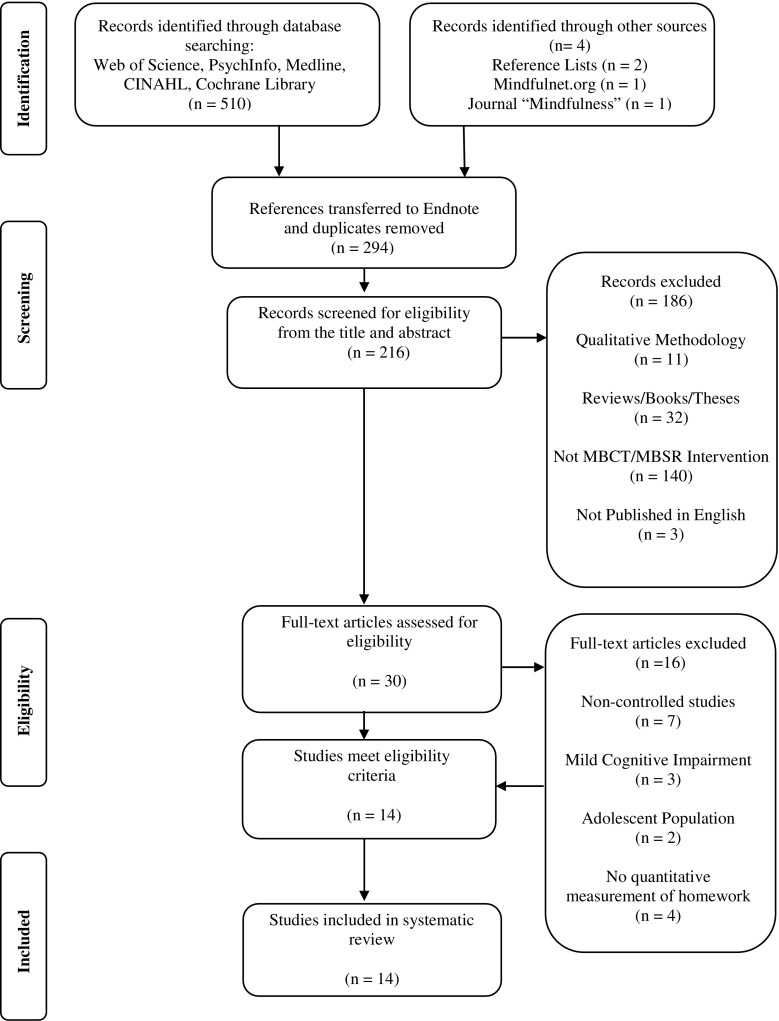
Flow diagram of selection of papers for inclusion in the systematic review

A study selection flow diagram is outlined in Fig. 1. The search strategy yielded a total of 514 articles. Search results from all five databases were exported to Endnote referencing software. Two hundred and ninety four studies remained after duplicates were removed. The titles and abstracts of these articles were screened for eligibility, which resulted in the exclusion of a further 186 studies. The full texts of the remaining 30 were reviewed; following which, 14 met all study eligibility criteria and were included in the final review.

### Quality Appraisal

The methodological rigour of each study was assessed using the Clinical Trials Assessment Measure (CTAM) (Tarrier and Wykes 2004). This 15-item measure was developed from the relevant features of the CONsolidated Standards of Reporting Trials (CONSORT) guidelines (Moher et al. 2001). The CTAM provides an overall representation of methodological rigour through ratings on six areas of trial design: sample size and recruitment method; allocation to treatment; assessment of outcome; control groups; description of treatments; and analysis (Lobban et al. 2013; Tarrier and Wykes 2004). Points are awarded for meeting quality standards on each of the subscales with a maximum score of 100. Wykes et al. (2008) proposed a CTAM score of 65 or above to indicate adequate methodology. Lobban et al. (2013) advised that studies should be compared based on subscales scores as a more appropriate appraisal as each category contributes a different weight to the overall score. The CTAM has shown adequate internal consistency and excellent concurrent validity (Wykes et al. 2008). To assess inter-rater reliability, an independent reviewer rated all 14 papers. Overall agreement was high and any discrepancies between reviewers were resolved through discussion.

## Results

### Description of Included Studies

A detailed description of the characteristics of included studies is shown in Table 1. This includes information on the study design, participant information, recruitment criteria, MBIs and control conditions, outcome and process measures utilised and the key findings. Overall, the studies examined a total of 725 participants. The median number of participants was 61.50 (interquartile range = 55). All studies were conducted in the developed world. Three studies (Bondolfi et al. 2010; Crane et al. 2014; Perich et al. 2013) were conducted in Europe and Australia and the remaining 11 studies were carried out in North America. The design of the studies included one secondary analysis of an RCT (Day et al. 2016), one study reported on data that was collected as part of an RCT of a mindfulness intervention (Crane et al. 2014), one study implemented a non-randomised controlled trial design (King et al. 2013) and the remaining 11 studies were RCTs. Six studies utilised MBCT and eight studies utilised MBSR. The durations of MBCT and MBSR were generally 8 weeks; however, two studies utilised an adapted MBI protocol of 7 weeks in duration with class time between 90 and 180 min per session (Johns et al. 2015; Speca et al. 2000). A wide range of outcome and process measures were used in studies including measures of psychological and physical functioning and measures of mindfulness.

**Table 1:** Characteristics and findings of included studies

Study and method	Participants	Recruitment	Intervention/conditions	Measures utilised	Key findings
Bondolfi et al. (2010) RCT **Country**: Switzerland	60 randomised, 43 females; 17 males MBCT + TAU median age = 46 years TAU median age = 49 years	History of major depressive disorder ≥ 3 episodes In remission and not taking medication	**MBCT + TAU**: 8 weekly × 2 h sessions, French translation MBCT manual utilised 4 MBCT booster sessions provided over 3 months follow-up **TAU**: seek treatment as normal	**Outcome**: SCID	Time to relapse was significantly longer for MBCT + TAU compared to TAU alone
Cash et al. (2015) RCT **Country**: USA	91 randomised, all female 18 years+	Diagnosis of fibromyalgia Females Available to attend weekly groups	**MBSR**: 8 weekly × 2.5 h sessions **Wait-list control**: offered the MBSR programme following study	**Outcome**: BDI CTQ PSS SSQ FSI FIQ	MBSR significantly reduced perceived stress, sleep disturbance and symptom severity, gains maintained at follow-up MBSR did not significantly alter pain, physical functioning or cortisol
Crane et al. (2014) RCT **Country**: UK	274 randomised, 198 females; 76 males Mean age of sample = 43 years, range 18–68 years	History of major depressive disorder ≥ 3 episodes Remission for the previous 8 weeks Informed consent from primary care physicians	**MBCT**: 8 weekly × 2 h session and 2 follow-up sessions at 6 weeks and 6 months post-treatment **Cognitive Psychological Education (CPE)**: 8 weekly × 2 h session and 2 follow-up sessions provided at 6 weeks and 6 months post-treatment **TAU**: seek treatment as normal	**Outcome**: SCID CTQ HAMD **Process**: MBI-TAC	See home-practice findings
Day et al. (2016) Secondary analysis of a RCT **Country**: USA	36 randomised, 32 females, 4 males Mean age of total sample = 41.7 years	19+ years old ≥ 3 pain days per month due to a primary headache pain If using medication, must have begun ≥ 4 weeks before baseline assessment	**MBCT**: 8 weekly × 2 h session and 2 follow-up sessions at 6 weeks and 6 months post-treatment, continued medical treatment as usual **Delayed treatment (DT)**: medical treatment as usual, then completed MBCT	**Outcome**: CSQ WAI-SF BPI CPEG **Process**: MBCT-AAQS	Therapists’ adherence and quality were both significant predictors of post-treatment client satisfaction Baseline pain intensity was positively associated with pre-treatment expectations, motivations and working alliance
Davidson et al. (2003) RCT **Country**: USA	41 randomised, 29 females, 12 males Average age of sample = 36 years, range = 23–56 years	Employees of Biotechnological Corporation in Madison, Wisconsin Right-handed	**MBSR**: 8 weekly × 2.5–3 h sessions, 7 h silent retreat **Wait-list control**: offered the MBSR programme following the study	**Outcome**: PANAS STAI	Meditation can produce increases in relative left-sided anterior activation that are associated with reductions in anxiety and negative affect and increases in positive affect
Dimidjian et al. (2016) Pilot RCT **Country**: USA	86 randomised MBCT-PD mean age = 31 years TAU mean age = 29 years	Pregnant adult women up to 32 weeks gestation History of major depressive disorder Available to attend weekly groups	**MBCT-PD**: adapted MBCT for peri-natal depression, 8 weekly × 2 h sessions, 1 monthly follow-up class **TAU**: free to continue or initiate mental health care	**Outcome**: SCID SCID-II CSQ LIFE EPDS	Significantly lower rates of relapse and depressive symptoms through 6 months post-partum in MBCT-PD compared to TAU MBCT-PD for at-risk pregnant women was acceptable based on rates of attendance and at-home-practice assignments
Gross et al. (2011) Pilot RCT **Country**: USA	30 randomised, 22 females, 8 males MBSR median age = 47 years PCT median age = 53.50 years	Diagnosis of primary insomnia Not taking sleep medication Adults English speaking	**MBSR**: 8 weekly × 2.5 h sessions and a day-long retreat (6 h) **Pharmacotherapy (PCT)**: 3 mg of eszopiclone nightly for 8 weeks and as needed for 3 months follow-up Plus 10 min presentation on sleep hygiene	**Outcome**: ISI PSQI DBAS-16 SSES STAI CES-D SF-12 **Other**: sleep diary	MBSR achieved reductions in insomnia symptoms and improvements in sleep quality comparable to PCT Higher treatment satisfaction in MBSR compared to PCT
Johns et al. (2015) Pilot RCT **Country**: USA	35 randomised, 33 females, 2 males MBSR-CRF mean age = 58.80 years Wait-list control mean age = 55.70 years	Diagnosis of cancer and clinically significant cancer-related fatigue (CRF) for 8 weeks 18+ years old	**MBSR-CRF**: 7 weekly × 2 h sessions and brief psycho-education on CRF, adapted MBSR for cancer-related fatigue **Wait-list control**: offered the MBSR programme following the study	**Outcome**: FSI SF-36 SDS PHQ-9 ISI PHQGADS	MBSR demonstrated significantly greater improvements in fatigue interference than controls and significant improvements in depression and sleep disturbance, improvements in symptoms maintained at 6-month follow-up MBSR proved acceptable to fatigued cancer survivors
King et al. (2013) Pilot non-randomised controlled trial **Country**: USA	37 participants MBCT mean age = 60.10 years TAU mean age = 58.30 years	Long-term >10 years PTSD or PTSD in partial remission All experienced combat-related traumas from military services	**MBCT**: adapted for combat-related PTSD, 8 weekly × 2 h sessions **TAU**: 8 × 1 h sessions of **Psychoed**: PTSD psycho-education and skills and **IRT**: 6 × 1.5 h sessions, of imagery rehearsal therapy	**Outcome**: PDS PTCI	MBCT proved an acceptable intervention for PTSD symptoms evidenced by engagement in programme and resulted in significant improvement in PTSD symptoms pre- vs post-MBCT compared to TAU and clinically meaningful improvement in PTSD symptom severity and cognitions
MacCoon et al. (2012) RCT **Country**: USA	63 randomised, 47 females, 16 males MBSR mean age = 44.50 years HEP mean age = 47.50 years	18–65 years Right-handed No previous experience of meditation English speaking In good general health	**MBSR**: 8 weekly × 2.5 h sessions, 7 h/day retreat **Health Enhancement Programme (HEP)**: 8 weekly × 2.5 h sessions, 7 hr day retreat, programme to match MBSR, activities valid active therapeutic ingredients but no mindfulness	**Outcome**: SCL-90-R MSC	Significant improvements for general distress, anxiety, hostility and medical symptoms but no differences between interventions, MBSR pain rating decrease compared to HEP HEP is an active control condition for MBCT
Perich et al. (2013) RCT **Country**: Australia	95 participants randomised, 62 females, 33 males No information on age provided	Diagnosis of bipolar I or II disorder, experienced 1+ episode over the past 18 months and lifetime of 3+ episodes Symptoms controlled on a mood stabiliser 18+ years of age, English speaking	**MBCT**: 8 weekly sessions, duration of each session not given. Followed Segal et al. (2002) protocol **TAU**: treatment as usual Both conditions received weekly psycho-educational material on bipolar disorder	**Outcome**: DASS STAI YMRS MADRS CIDI SCID **Process**: MAAS TMS	See home-practice findings
Speca et al. (2000) RCT **Country**: Canada	90 randomised, 73 females, 17 males Mean age of sample = 51 years, age range = 27–75 years	Diagnosis of cancer at any time point were eligible to participate	**MBSR**: 7 weekly × 1.5 h sessions, adapted version of Kabat-Zinn MBSR programme **Wait-list control**: offered the MBSR programme following the study	**Outcome**: POMS SOSI	MBSR effectively reduced mood disturbance, fatigue and a broad spectrum of stress-related symptoms
Wells et al. (2014) Pilot RCT **Country**: USA	19 randomised, 17 females, 2 males MBSR mean age = 45.90 years TAU mean age = 45.20 years	Diagnosis of migraine, ≥ 1 year history of migraines Available to attend weekly sessions 18+ years old English speaking	**MBSR**: 8 weekly × 2 h sessions plus 1-day (6 h) retreat. Utilised Kabat-Zinn protocol **TAU**: continue with care as usual and asked not to start a yoga or meditation during study. Offered MBSR following the study	**Outcome**: HIT-6 MIDAS MSQ PHQ-9 STAI PSS-10 HMSES **Process**: FFMQ	MBSR is safe and feasible for adults with migraines Secondary outcomes demonstrated that MBSR had a beneficial effect on headache duration, disability, self-efficacy and mindfulness
Whitebird et al. (2012) RCT **Country**: USA	78 randomised, 69 females, 9 males MBSR mean age = 56.40 years CCES mean age = 57.20 years	Self-identified as primary caregiver of family member with dementia 21+ years old English speaking	**MBSR**: 8 weekly × 2.5 h sessions, 5-h day retreat **Community Caregiver Education Support (CCES)**: 8 weekly × 2.5 h sessions, 5-h retreat day. Education on issues affecting family caregivers and group social and emotional support	**Outcome**: PSS CES-D STAI SF-12 MBCBS MOSSSS	MBSR is a feasible and acceptable intervention for dementia caregivers, MBSR improved overall mental health, reduced stress and decreased depression at post-intervention compared to CCES Both interventions improved caregiver mental health, anxiety, social support and burden

*SCID* Structured Clinical Interview for DSM-IV (First et al. 1996), *SSQ* Stanford Sleep Questionnaire (Douglass et al. 1994), *BDI* Beck Depression Inventory (Beck et al. 1961), *FSI* The Fatigue Symptom Inventory (Hann et al. 1998), *CTQ* Childhood Trauma Questionnaire (Bernstein and Fink 1998), *FIQ* Fibromyalgia Impact Questionnaire (Burckhardt et al. 1991), *PSS* Perceived Stress Scale (Cohen et al. 1983), *CSQ* Client Satisfaction Questionnaire (Attkisson and Zwick 1982), *WAI-SF* Working Alliance Inventory-Short Form (Hatcher and Gillaspy 2006), *HAMD* Hamilton Rating Scale for Depression (Hamilton 1960), *BPI* Wisconsin Brief Pain Inventory (Cleeland and Ryan 1991), *MBI-TAC* Mindfulness-Based Interventions-Teaching Assessment Criteria Scale (Crane et al. 2013), *MBCT-AAQS* MBCT Adherence, Appropriateness and Quality Scale (Day et al. 2014), *CPEG* Checklist of Patient Engagement in Group Form (Mignogna et al. 2007), *PANAS* Positive and Negative Affect Schedule (Watson et al. 1988), *EPDS* Edinburgh Post-partum Depression Scale (Cox et al. 1987), *STAI* State-Trait Anxiety Inventory (Spielberger et al. 1970), *ISI* Insomnia Severity Index (Bastien et al. 2001), *PSQI* Pittsburgh Sleep Quality Index (Buysse et al. 1989), *SCID-II* Structured Clinical Interview for DSM-IV Axis II Personality Disorders (First et al. 1997), *DBAS-16* Dysfunctional Beliefs and Attitudes about Sleep (Morin et al. 2007), *SSES* Sleep Self-Efficacy Scale (Lacks 1987), *LIFE* Longitudinal Interval Follow-up Evaluation (Keller et al. 1987), *CES-D* Centre for Epidemiological Studies Depression Scale (Radloff 1977), *SF-12* Short-Form 12 Item Health Survey (Ware et al. 1996), *PDS* PTSD Diagnostic Scale (Foa et al. 1997), *SF*-36 Medical Outcomes Study 36-item Health Survey (Ware & Sherbourne, 1992), *PTCI* Post-traumatic Cognitions Inventory (Foa et al. 1999), *SDS* Sheehan Disability Scale (Sheehan et al. 1996), *PHQ-9* Patient Health Questionnaire (Kroenke and Spitzer 2002), *PHQGADS* Patient Health Questionnaire Generalised Anxiety Disorder (Spitzer et al. 2006), *SCL-90-R* Symptom Checklist-90-Revised (Derogatis 1996), *MADRS* Montgomery-Asberg Depression Rating Scale (Montgomery and Asberg 1979), *MSC* Medical Symptoms Checklist (Travis and Ryan 1977), *CIDI* Composite International Diagnostic Interview (Kessler et al. 1998), *SCID* Structured Clinical Interview for DSM-IV (First et al. 1996), *MASS* Mindfulness Attention Awareness Scale (Brown and Ryan 2003), *DASS* Depression Anxiety Stress Scale (Lovibond and Lovibond 1993), *TMS* Toronto Mindfulness Scale (Lau et al. 2006), *POMS* Profile of Mood States (McNair et al. 1992), *YMRS* Young Mania Rating Scale (Young et al. 1978), *SOSI* Symptoms of Stress Inventory (Leckie and Thompson 1979), *HIT-6* Headache Impact Test-6 (Kosinski et al. 2003), *FFMQ* Five Facets Mindfulness Questionnaire (Baer et al. 2006), *MIDAS* Migraine Disability Assessment (Stewart et al. 1999), *MSQ* Migraine Specific Quality of Life Questionnaire (Jhingran et al. 1998), *SF-12* Short-Form 12 Item Health Survey (Ware et al. 1996), *PSS-10* Perceived Stress Scale (Cohen et al. 1983), *MBCBS* Montgomery Borgatta Caregiver Burden Scale (Montgomery et al. 2000), *HMSES* Headache Management Self-Efficacy Scale (French et al. 2000), *MOSSSS* Medical Outcomes Study Social Support Survey (Sherbourne and Stewart 1991)

### Methodological Quality

Table 2 provides CTAM subscale and total scores for each of the 14 studies reviewed. CTAM total scores varied widely ranging from 30 to 84 (median = 53.50, interquartile range = 16). Only four studies (Bondolfi et al. 2010; Crane et al. 2014; Dimidjian et al. 2016; Perich et al. 2013) achieved a CTAM total score equal to or greater than the arbitrary cutoff of 65 as suggested by Wykes et al. (2008), indicating adequate methodological quality. There was variability in methodology, with many limitations across studies resulting in low scores being allocated. Six studies scored full marks on the sample subscale utilising a geographic cohort and sufficient sample size. All studies except one (King et al. 2013) had random allocation; however, the process of randomisation was not always described or carried out independently from the trial research team. Generally poor scores were designated for the ‘assessment’ subscale due to a lack of blinding and poor descriptions of blinding procedures. With regards to control groups, eight studies utilised TAU or wait-list control groups and therefore non-specific treatment effects could not be controlled for, contributing to a poor rating on this subscale. All studies employed statistical methods deemed appropriate for the outcome measure, and ten studies conducted intent-to-treat analysis. Finally, the delivery of treatment was guided by a treatment protocol for all studies except two (MacCoon et al. 2012; Whitebird et al. 2012), but for 8 of the 14 studies adherence to the treatment protocol or treatment quality was not assessed.

**Table 2:** CTAM subscale scores

Study	Sample (10)	Allocation (16)	Assessment (32)	Control groups (16)	Analysis (15)	Active treatment (11)	Total (100)
Perich et al. (2013)	10	16	26	6	15	11	84
Bondolfi et al. (2010)	10	16	26	6	15	8	81
Crane et al. (2014)	10	16	6	16	9	11	68
Dimidjian et al. (2016)	7	10	16	6	15	11	65
MacCoon et al. (2012)	5	16	16	10	15	0	62
Gross et al. (2011)	10	16	6	10	9	3	54
Whitebird et al. (2012)	10	13	6	10	15	0	54
Day et al. (2016)	5	13	6	6	15	8	53
Cash et al. (2015)	10	16	6	0	15	3	50
King et al. (2013)	2	0	6	16	15	8	47
Speca et al. (2000)	7	13	6	0	15	6	47
Wells et al. (2014)	2	10	6	6	15	6	45
Johns et al. (2015)	2	13	6	0	9	3	33
Davidson et al. (2003)	2	10	6	0	9	3	30

### Home-Practice Characteristics

Table 3 outlines the monitoring, guidance, reporting and findings related to home-practice across studies. This table includes some of the more detailed results of this review and complements the main findings. Therefore it should be referred to in addition to the narrative synthesis.

**Table 3:** Home-practice characteristics

Study	Guidance for home-practice	Resources given to participants	Measurement of home-practice	Total reported practice	Proportion of recommended practice achieved	Home-practice findings
Bondolfi et al. (2010)	Frequency of practice not specified	2 CDs with recordings of body scan, sitting meditation, mindful movement and 3-min breathing space	Retrospective ad hoc self-report questionnaire	**% practice once per week**: Body scan = 65.4% Sitting meditation = 88% 3-min breathing = 91.7% Informal practice = 76%	Could not be calculated	Amount of home-practice did not significantly differ between those who relapsed and those who did not (Fisher’s exact test, N.S.) Following treatment the frequency of informal home-practice remained unchanged over 14 months but longer formal meditation practice decreased over time
Cash et al. (2015)	45 min × 6 days a week, practice of body scan, sitting meditation, yoga positions	Workbook and audio-tapes of mindfulness exercises	Self-report weekly log of home-practice and qualitative assessment of how much practice completing at follow-up	Reported practice 4.8 times per week at 2-month follow-up	Could not be calculated	Greater home-practice at follow-up was associated with reduced pain (*R* ^2^ = 0.42; *p* < 0.01, partial *r* = −0.45) and symptom severity of fibromyalgia (*R* ^2^ = 0.24; *p* < 0.05, partial *r* = −0.40)
Crane et al. (2014)	40 min × 6 days a week, both formal and informal practices required	CD of formal mindfulness exercises	Self-report weekly diary of home-practice	Reported formal practices on average 3.36 days per week, average duration was 21.31 min. Mean no. of units of informal practice was 80.44 over treatment	26.51%	A significant positive association between mean daily duration of formal home-practice and outcome in MBCT was found. Those who practiced on an average of 3 or more days per week were approximately half as likely to relapse to depression over 12 months of follow-up as those who practiced less frequently [*B* = −0.03, SE = 0.013, Wald (1) = 5.51, *p* = 0.018, HR = 0.97, Cl = 0.947 to 0.995] No association between amount of informal home-practice and time to relapse was found [*B* = −0.002 (SE = 0.002), Wald 1.74, *p* = 0.19, HR = 1.00, Cl = 0.99 to 1.00]
Day et al. (2016)	45 min × 6 days a week, practice	No information noted	Self-report daily meditation practice diary (online administration)	Reported a mean total of 21.69 h of practice throughout MBCT programme	60.25%	In-session engagement significantly positively predicted client attendance (*β* = 0.454; *R* ^2^ = 0.207; *F*_1,19_ = 4.945; *p* = 0.038; power = 0.6) and time spent in at-home meditation practice throughout treatment (*β* = 0.482; *R* ^2^ = 0.232; *F*_1, 19_ = 5.749; *p* = 0.027; power = 0.7). Fidelity ratings were not associated with amount of home-practice (*p* > 0.05)
Davidson et al. (2003)	Assigned formal and informal practices 1 h × 6 days a week	Guided audio-tapes to guide mindfulness practices	Self-report daily log of the frequency, number of minutes and techniques of formal meditation practice	Reported mean practice on 2.48 days out of 6 and mean practice 16.19 min per time after intervention, after 4 month follow-up reported mean practice on 1.70 days out of 6 and mean practice 14.21 min per time	14.87%	There were no significant associations between the measures of practice and brain activity or biological or self-report measures
Dimidjian et al. (2016)	Specific practices assigned for 6 days each week but amount of time not specifically reported	Audio-files to guide mindfulness practices and a DVD to guide yoga practice	Self-report weekly log of no. of times and type of home-practice	67% provided practice data, on average practicing 30 out of the 42 assigned days, with a higher total frequency of informal practice than formal practice	Could not be calculated	None reported
Gross et al. (2011)	45 min of meditation × 6 days a week for 8 weeks and 20 min daily for 3 months follow-up	Audio-files of recorded meditations and handouts of assignments	Tracked electronically using a pocket size logger which participants turned on every time they began a meditation	17 patients reported practice data mean 23.7 min per day during intervention and 16 participants reported 21.8 min per day during follow-up	61.44%	Reductions in DBAS-16 and activity limitation due to insomnia scores were significantly predicted by home-practice during intervention period (Spearman’s rho correlations = 0.62 and 0.71, *p*s < 0.02)
Johns et al. (2015)	20 min practice of body scan, sitting meditation and yoga, no specific guidance reported on number of days per week to practice	Audio-recordings of guided meditations. Participants received $5 for each weekly log submitted	Self-report weekly log of home-practice minutes per day and type of practice	16/18 submitted practice logs every week, average 35 min practice per day during programme, 6 month follow-up 20 min formal practice on 2 days and informal practice on 3.8 days per week	45.37%	None reported
King et al. (2013)	15–20 min of formal and informal practice 5 days a week, guidance on informal practice given	Received audio-files of formal mindfulness exercises	Self-report weekly log of home-practice minutes per day and what recordings they had listened to	Reported on average 102.3 min of formal practice per week and 12.2 additional minutes of informal practice on days practice was reported	37.88%	None reported
MacCoon et al. (2012)	45 min practice 6 days a week, no guidance on what exercises to practice reported	None reported	Self-report weekly log of minutes and sessions of informal home-practice during the MBSR programme and for the 4 month follow-up period	Average 1849 min of practice reported (44 min over 6 days), average 4394 min of practice reported during 4 month follow-up period (25 min 6 days a week)	85.6%	Home-practice was not related to change in outcome measures for pain or psychological distress (*R* ^2^s ≤ 0.06, *p* > 0.05)
Perich et al. (2013)	Formal practice for 5 weeks of programme was 40 min body scan or sitting meditation with CD and 2 weeks without aid of CD for 30–40 min	Received audio-files of formal mindfulness exercises	Self-report weekly log of daily practice. Recorded whether they had engaged in practicing particular exercises, did not measure time spent practicing	67% provided practice data, mean number of days engaged in at least 1 meditation practice per day was 26.4 days (range 5–44 days) during MBCT programme. 13 noted to continue practice at 12-month follow-up	Could not be calculated	The number of prior bipolar episodes was negatively correlated with number of days practicing [*r*(23) = −0.512, *p* = 0.013]. Number of days practicing was not significantly correlated with any of the post-treatment symptoms scores A greater no. of days practicing during the MBCT programme was negatively correlated with depression scores at 12-month follow-up [*r*(16) = −0.559, *p* = 0.024] Evidence to suggest that practice was associated with improvements in depression and anxiety symptoms if a minimum of 3 days a week practice was completed during MBCT programme
Speca et al. (2000)	Specific weekly guidance on what exercises to practice reported but no information on the duration of practice or how many days a week to practice was stated	Received workbook and audio-tape of guided meditation	Self-report record form of duration of participant’s daily meditation practice	Average total daily practice MBSR group during programme was 32 min	82.96%	Number of minutes spent engaging in home-practice significantly predicted POMS change scores [*F*(2, 43) = 3.94, *p* < 0.03] and accounted for 15.5% of the variance in mood improvement. Number of minutes of practice significantly predicted changes in total mood disturbance [*r*(81) = 2.73, *p* < 0.01]
Wells et al. (2014)	45 min per day, 5 days a week	Given guided audio-recordings to follow during practice	Self-report daily logs of home-practice	Daily meditation average 34 ± 11 min, range 16–50 min per day	88.14%	None reported
Whitebird et al. (2012)	No specific guidance reported	Given CDs and written material of home-practice	Self-report measure of minutes per day practice in health behaviour calendars	Reported an average of 6.8 sessions of practice per week and averaged 29.4 min per session during the MBSR programme	74.04%	None reported

### Home-Practice Monitoring

All 14 studies utilised self-report measures to monitor home-practice for both formal and informal practices. The majority of studies utilised self-report logs, diaries, questionnaires or calendars to monitor practice. One study (Gross et al. 2011) used an electronic device (logger) to track the length of their home-practice. The logger was a pocketsize, battery-operated recording device, which stores a date/time stamp whenever it was switched on or off. Cash et al. (2015) used both a log and a retrospective qualitative report of the number of times practiced per week at the end of each assessment period. Day et al. (2016) was the only study to administer their log of home-practice via an online portal. Johns et al. (2015) gave a financial incentive ($5 for each weekly log) to participants to monitor their home-practice. With respect to monitoring of home-practice frequency and duration, the majority of studies monitored practice specifying the amount of minutes practiced per day or the frequency of times practiced per week. No study reported on the psychometric properties of the monitoring methods nor included the log/diary in the appendices of the study. Overall, these findings illustrate the wide variation in how studies measure home-practice compliance and suggest that at present there is no evidenced based manner in which to do so across MBI studies.

### Guidance and Resources for Home-Practice

Studies were reviewed for the guidance and resources given to participants for their home-practice across the MBIs. The formal practices noted across studies included sitting meditation, body scan meditation, 3-min breathing space, mindful movement and mindful yoga practices. Informal practices were not outlined in the majority of studies but suggestions such as mindfulness of routine activities and bringing mindful awareness to moments in daily life were reported. Of the eight MBSR studies included in this review, only four studies (Cash et al. 2015; Davidson et al. 2003; Gross et al. 2011; MacCoon et al. 2012) outlined formal home-practices exactly in accordance to the MBSR recommendations of 45 min × 6 days a week. However, only Davidson et al. (2003) noted both the formal and the recommended 5–15 min informal practice in their guidance. One study (Johns et al. 2015) adapted their home-practice tasks for a cancer context and therefore reduced the amount of practice to 20 min sessions. Of the six MBCT studies, only half (Crane et al. 2014; Day et al. 2016; Perich et al. 2013) outlined home-practice in accordance to the MBCT recommendations of 45 min × 6 days a week. King et al. (2013) adapted their guidance to 15–20 min of formal and informal practice 5 days a week for participants with combat-related fatigue.

With respect to home-practice resources, two studies (Day et al. 2016; MacCoon et al. 2012) did not indicate if resources were provided. Across the other 12 studies, participants were given audio-recordings, CDs or audio-tapes of formal mindfulness exercises to utilise for home-practice. Additional resources noted across some studies included workbooks or written material and a DVD to complete their yoga exercises. These findings illustrate that the guidance on the length of home-practice was variable across studies, which indicates that research studies and teachers are not adhering to the published protocols. In addition, there was also variability in the resources given to participants. Crane et al. (2017) advocate for MBI titles only to be utilised in research when the MBCT/MBSR protocol are being followed.

### Amounts of Home-Practice Reported Across Studies

As outlined in Table 3, all studies reported the amounts of home-practice that participants engaged in throughout treatment except Cash et al. (2015) who measured home-practice during treatment but only reported it at follow-up. There was inconsistency in how the quantity of the home-practice was reported. The length and frequency of practice were reported in seven studies (Davidson et al. 2003; Gross et al. 2011; Johns et al. 2015; MacCoon et al. 2012; Speca et al. 2000; Wells et al. 2014; Whitebird et al. 2012) ranging from 16.9 min on 2.48 days out of six (Gross et al. 2011) to 44 min six days a week (MacCoon et al. 2012). A number of studies divided amounts of practice into formal and informal mindfulness practice. This ranged from formal meditation practice on 3.36 days a week for 21.31 min and a mean of 80.44 times of informal practice throughout treatment (Crane et al. 2014) to 102.3 min per week of formal meditation and an additional 12.2 min of informal meditation per day (King et al. 2013). None of the included studies noted the overall completion rates of home-practice diaries by participants.

### Maintaining Home-Practice Post-Intervention

Post-intervention home-practice was reported in six studies. Documented practice in these studies ranged from 14.21 min per session on 1.70 days out of 6 (Davidson et al. 2003) to 25 min six days a week (MacCoon et al. 2012) over follow-up periods of 4 and 5 months (Gross et al. 2011). Four of these studies (Bondolfi et al. 2010; Cash et al. 2015; Johns et al. 2015; Perich et al. 2013) reported the maintenance of practice as frequencies per week over follow-up periods of 2, 6, 7–12 and 12 months. These findings indicate that the included studies varied extensively in how they reported home-practice during treatment and post-intervention. None of the included studies had an active control which measured home-practice as a comparison to MBI home-practice.

## Amount of Home-Practice and MBSR/MBCT Guidelines

It was possible to calculate the mean values for duration of formal home-practice in the studies as a percentage of the durations recommended for MBI. This was calculated by determining the total amount of practice reported over 6 days per week in each study and expressing this as a percentage of the recommended 45 min × 6 days a week (270 min) outlined in the MBSR/MBCT recommendations. Table 3 outlines the percentages across all studies these ranged from 14.87% (Davidson et al. 2003) to 88.14% (Wells et al. 2014). For the remaining four studies (Bondolfi et al. 2010; Cash et al. 2015; Dimidjian et al. 2016; Perich et al. 2013), it was not possible to calculate the percentage of formal home-practice expectations met as these studies did not report home-practice in minutes. It was not feasible to determine the percentage of the informal practice expectations that were achieved in studies, as the majority of studies did not report the amount of informal practice that participants engaged in.

## Associations of Home-Practice and Clinical Outcomes

As outlined in Table 3, seven studies examined the relationship between amount of home-practice and measures of clinical outcome. In all of the included studies, these results were secondary as opposed to primary analyses of outcomes. Of these, four studies (Cash et al. 2015; Crane et al. 2014; Gross et al. 2011; Speca et al. 2000) demonstrated amounts of home-practice predicted improvements on clinical outcome measures, the other three studies did not find a significant effect of practice on clinical measures. Crane et al. (2014) reported that participants who practiced on three or more days a week were almost half as likely to relapse to depression as those who practiced less frequently. However, Bondolfi et al. (2010) found that amounts of home-practice did not differ between those who relapsed to depression (*n* = 9) and those who did not relapse (*n* = 17) (both measured by the SCID (First et al. 1996)). Perich et al. (2013) found no association between number of days practice and outcome measures following treatment or at 12-month follow-up. They found those who practiced a minimum of once a day for 3 days a week compared to 2 days a week or less resulted in significant differences in anxiety scores (STAI; Spielberger et al. 1970) and lower scores on depression outcomes (DASS; Lovibond and Lovibond 1993). Furthermore, at 12-month follow-up, participants who practiced more frequently during treatment had significantly lower depression scores.

Three studies (Crane et al. 2014; Day et al. 2016; Perich et al. 2013) examined home-practice with measures other than clinical outcomes. Day et al. (2016) reported that participants with higher in-session engagement (teacher-rated) spent a greater amount of time practicing. However, they reported that fidelity to protocol ratings (measured by MBCT Adherence, Appropriateness and Quality Scale; Day et al. 2014) were not associated with amounts of home-practice. Crane et al. (2014) found no relationship between treatment plausibility (idiosyncratic measure) and home-practice. Finally, Perich et al. (2013) was the only study to measure the relationship between home-practice and levels of mindfulness but found no significant differences in mindfulness (as measured by Mindfulness Attention Awareness Scale; Brown and Ryan 2003) between those who continued home-practice at 12-month follow-up and those who did not. The remaining five studies (Dimidjian et al. 2016; Johns et al. 2015; King et al. 2013; Wells et al. 2014; Whitebird et al. 2012) did not evaluate the relationship between home-practice and clinical outcomes or other measures. These studies reported amounts of practice as an aspect of adherence, feasibility, acceptability and satisfaction or compliance and retention to treatment.

## Discussion

One aspect of MBIs posited to be important in increasing the therapeutic effects of the intervention is participants’ engagement in regular home-practice. Despite this, the research findings evaluating home-practice and clinical outcomes are mixed (Vettese et al. 2009). To date, there has been a small volume of systematic reviews conducted in this area but no review of controlled MBI studies and home-practice. Therefore, this review examined available controlled group MBI literature that measured home-practice utilising a self-report measure. Fourteen studies that investigated associations between home-practice and a range of outcome measures were included in this review.

A key aim of the review was to explore how home-practice was measured across different evaluations of MBIs. There was wide variety in the methods utilised to monitor practice from an electric logger (Gross et al. 2011) to home-practice logs/diaries (e.g. Cash et al. 2015). There was limited information provided regarding the content of the measurements or how they were developed. The inconsistency in the monitoring of home-practice compliance is reflected in the data that these tools produced, which restricted meaningful interpretation of compliance rates across studies. All studies focussed on the monitoring the *quantity* of home-practice rather than exploring ways of assessing and/or maximising the *quality* of this home-practice. The total duration of mindfulness practice has been hypothesised to be important for positive outcomes. However, adherence involves not only attempting the practice but also adhering to the specific way in which mindfulness practices should be conducted (e.g. present moment, non-judgemental attention). Therefore, quality of practice could be an important factor for predicting outcomes. One such tool that has been developed is the Practice Quality-Mindfulness (PQ-M; Del Re et al. 2013), which could be implemented in studies. The PQ-M is a six-item self-report measure that is utilised as a tool for assessing changes in mindfulness practice quality over time. These findings indicate that there is a need for the development of greater sophistication and consistency in methods being employed to monitor home-practice across MBIs. These measures need to monitor the level to which home-practice corresponds to the guidelines of MBSR and MBCT, measuring both the minutes and frequency of formal and informal practice.

Another important consideration for this review was the home-practice resources and guidance given to participants. The resources were varied but the majority of studies gave participants audio-recordings to enable guided home-practice of formal exercises. Research is needed to determine what specific resources increase engagement in home-practice. This review demonstrated that the majority of studies gave participants practice guidance that is approximately in line with MBI recommendations. Six studies did not give the specific details regarding duration of practice or adapted the recommended practice guidelines for the population completing the intervention. This discrepancy between what is recommended and what is reported on home-practice in studies further contextualises the mixed findings on home-practice and its relationship to clinical outcomes. It may be that facilitating participants to engage better in home-practice could strengthen the relationship between practice and clinical outcomes. Additionally, it could be hypothesised that individual teacher factors will have a significant impact on adherence to home-practice. The subtlety of how teachers motivate their participants to engage in home-practice may play an important role in adherence to practice and subsequently outcomes for MBIs. Therefore, assessing the competence and adherence of mindfulness class-based teaching could be important to addressing barriers to engagement in practice. The Mindfulness-Based Interventions Teaching Assessment Criteria (MBI-TAC; Crane et al. 2013) is an assessment tool, which covers six domains of the teaching process to assess mindfulness-based teacher competence. Future research could investigate whether high scores on certain domains of the MBI-TAC are correlated with increased home-practice engagement.

The current review, as with the review conducted by Parsons et al. (2017), found that participants’ practice reports were variable both within individual studies and across different studies. Despite these indications that participants struggle to complete the stipulated amount of home-practice guidance, none of the studies included in the current review explored the barriers that participants experienced. This is an important aspect that has been relatively overlooked in mindfulness research. In terms of cognitive behavioural therapy (CBT), Dunn et al. (2002) found that factors such as motivation, recall of the assignment, difficulty, understanding of the rationale, perceived benefits and effort affected home-practice compliance. MBSR and MBCT stipulate home-practice that requires significant time commitments from participants, which may impact on their engagement and motivation. It is important that the barriers and individual-level factors affecting completion of home-practice are explored in the context of MBI to help maximise the efficacy of the interventions. The studies in this review included a range of populations such as individuals with major depressive disorder (Bondolfi et al. 2010) and participants diagnosed with bipolar disorder (Perich et al. 2013). It is important that the impacts of these enduring mental health difficulties along with other physical and somatic conditions are taken into consideration when evaluating the amounts of home-practice reported in trials with these populations.

Despite home-practice being hypothesised as an important factor for outcomes in MBI, only a small sample of studies in this review have investigated the relationship between home-practice and clinical outcomes. Of the included studies only half examined this relationship, of which four studies demonstrated a significant effect. These studies focused on a range of outcomes, both psychological and physical health, and analysed this relationship using a variety of statistical methods. In addition, only one included study examined the relationship between practice amounts and levels of mindfulness (as assessed by the MAAS; Brown and Ryan 2003) in participants. These findings raise a number of criticisms of evaluations of MBIs that are similar to the following ones by Vettese et al. (2009). Of the studies that investigated the relationship between practice and clinical outcomes, most studies regarded the mindfulness practice component as a secondary rather than a primary focus of the research and the number of studies investigating the association between practice and levels of mindfulness is limited. However, the Parsons et al. (2017) review identified 48 studies, which reported formal home mindfulness practice data. This illustrates an increase in the volume of research over the last decade investigating home-practice, including its relationship with clinical outcomes. Parsons et al. (2017) found a small significant association between participants’ home-practice and clinical outcomes. It is key that future research routinely investigates whether duration of home-practice increases levels of mindfulness, as this is posited to subsequently improve the therapeutic effects of the intervention (Kabat-Zinn 2013).

Dimidjian and Segal’s (2016) review of MBI research highlights teacher factors and implementation questions as a critical area for the MBI research agenda going forward. In terms of mindfulness home-practice, this review recommends further RCTs that experimentally manipulate the dose of home-practice to assess differential effects. There has been mixed findings regarding whether the use of comparatively small ‘doses’ of mindfulness practices, relative to those prescribed by MBSR and MBCT, can result in positive clinical outcomes. Howarth et al. (2016) found that a brief mindfulness intervention was well accepted among patients with long-term illness (i.e. chronic pain, cardiovascular disease), and they reported improved coping with symptoms. MacKenzie et al. (2006) found that following a brief 4-week MBSR intervention resulted in participants experiencing significant improvements in burnout symptoms, relaxation and life satisfaction. However, a recent study by Reynolds et al. (2017) reported increased symptom distress, social avoidance and reduced quality of life among cancer patients following a brief mindfulness intervention.

Although MBIs recommend both formal and informal practice, the included studies focused on the relationship between formal mindfulness practice and clinical outcomes. The effects of informal practice are under-examined. A number of studies have failed to find a direct relationship between informal mindfulness practice and associated changes on clinical measures (Carmody and Baer 2008; Hawley et al. 2014). This may be as a result of the nature of informal practice, which is more challenging to isolate and therefore it is hard to measure the frequency and duration of this practice. Improved methods of monitoring this type of practice, such as experience sampling, may be valuable in future research. Additionally, it could be that the actual amount of formal home-practice is not as important for clinical outcomes as participants’ informal exploration and use of techniques in their everyday lives.

## Limitations and Recommendations

There are a number of limitations that should be taken into account when considering the conclusions of this review. Firstly, limitations of the use of the CTAM (Tarrier and Wykes 2004) as an assessment of methodological quality must be acknowledged. The CTAM has been used to assess the methodological quality in a number of reviews (Wykes et al. 2008) and has shown good blind inter-rater agreement, adequate internal consistency and excellent concurrent validity with other established rating scales designed to assess the generic quality of clinical trials (Lobban et al. 2013). That said, other tools such as The Cochrane Collaborations Risk of Bias Tool (2011) are supported by PRISMA-P guidelines, which emphasize additional domains that may need to be considered when evaluating RCTs (Lobban et al. 2013). However, the use of the CTAM in the current review provides a different perspective on methodological rigour to the review conducted by Parsons et al. (2017), which assessed risk of bias across MBI studies. Secondly, the heterogeneity of the included studies such as study sample selection; outcome measures utilised; home-practice measurement and guidance and the range of presenting problems across studies, made direct comparisons of home-practice between studies, challenging. Additionally, there was a lack of inter-rater reliability in the process of screening the abstracts for inclusion, as not all abstracts were second-screened by an independent evaluator. This may mean a small number of studies, which met inclusion criteria, were missed.

Thirdly, there are limitations regarding the scope of this review, which included a small number of studies. Studies that have measured home-practice in other ways (e.g. qualitative methods of enquiring about home-practice during and post-treatment) and non-controlled studies, of which there are a number of recent studies examining home-practice in MBI, were excluded. Additionally, two included studies (Johns et al. 2015; Speca et al. 2000) used adapted protocols of MBIs of 7 weeks in duration. These studies should be interpreted with caution as they are potentially delivering protocols that vary from the core structure, form, dose and delivery method of traditional MBIs. A need for standardisation in how MBIs are administered and ensuring that participants receive an adequate ‘dose’ will be important for efforts aimed at determining the efficacy of MBIs (Crane et al. 2017). Finally, it is important to highlight the difficulties associated with the measurement of home-practice and the impact of this on the outcomes of MBIs. The majority of studies utilise self-report measures to monitor home-practice. Given the subjective nature of this type of measurement, there is no reliable way to ensure that this practice has occurred. Therefore, it is difficult to reliably draw conclusions regarding the relationship between the amount of home-practice completed and whether this improves MBI outcomes or not.

As a result of this review, a number of recommendations can be made that will serve to enhance future research on the efficacy of home-practice in group-MBI. It is evident from the appraisal of this research that the majority of studies have been conducted in North America and Europe. It is important that future MBI research is conducted in other areas of the world, to develop findings that can be generalised to wider populations. The findings illustrate the need for mindfulness research more generally to utilise experimental methodologies consistently to allow for firm conclusions about the effects of home-practice on clinical outcomes. It is imperative that future research explores the amount of home-practice across populations; barriers and motivators to home-practice; and that cumulative rather than average estimates of practice are used to elucidate the role of home-practice in MBIs. This review illustrates the need for the development of more standardised measures for monitoring the quantity of practice. This would allow for consistency in how home-practice is measured across different studies and hence the comparison of findings across these studies. With this in mind, the authors of the current review have developed the *Mindfulness Home-Practice Monitoring Form* (*MHMF*), a measurement tool that could be utilised to monitor formal and informal home-practice in future MBI studies. The MHMF (see Table 4) is a self-report measure that monitors both the length and frequency of formal and informal mindfulness practice, resources used for practice and any barriers encountered by participants. This measure was developed on the basis of the findings of this review, which highlighted the need for a standardised method of monitoring home-practice across MBIs.

**Table 4:** Mindfulness Home-Practice Monitoring Form (MHMF)

Formal practice				
Day and Date	✓ Practiced	Practices Completed (Minutes Practicing)	Resources Used	Comments/Barriers to Practice
Monday Date:	Ex. ✓Yes	Sitting Meditation (20 min) Body Scan (20 min)	Mindfulness CD	
Tuesday Date:				
Wednesday Date:				
Thursday Date:				
Friday Date:				
Saturday Date:				
Sunday Date:				
**Informal practice**				
Day and Date	✓ Practiced	Minutes Practicing	Activities Completed	Comments/Barriers to Practice
Monday Date:	Ex. ✓Yes	20 min	Mindfulness during washing dishes	
Tuesday Date:				
Wednesday Date:				
Thursday Date:				
Friday Date:				
Saturday Date:				
Sunday Date:				

Another important consideration moving forward will be developing techniques for assessing the *quality* of home-practice. Qualitative research and methods of exploring home-practice including the exploration of the barriers participants’ experience in completing home-practice could additionally help inform ways to facilitate better compliance. In addition, Experience Sampling Methodology (ESM)/Ecological Momentary Assessment (EMA) will provide important opportunities for the quality and quantity of the mindful orientation that research participants adopt in their daily lives. Important opportunities exist for using mobile technology (e.g. mobile phone apps) to be used for the real-time monitoring of mindfulness levels between sessions. Parsons et al. (2017) review findings also advocate for the use of mobile technology in future research. The affordability and the near ubiquity of mobile phones will make it easier to scale interventions and enrich assessment and research with contextual data about functioning in daily life. Clinicians can make use of mobile technologies in a variety of ways in MBIs. Many apps exist that include resources and formal mindfulness practice recordings that can be utilised to supplement home-practice during the intervention and for maintenance of practice after the intervention has ended (e.g. Mindfulness, NHS Greater Glasgow and Clyde). In addition, the use of mobile technology to record real-time mindfulness practice and text reminders to complete home-practice could make practice more accessible for participants, particularly if they are additionally using mobile technology to listen to recordings of formal practices. Therefore, this could increase both the amount of home-practice completed and the richness of the data on home-practice. In addition to monitoring via self-report apps, a variety of apps use data from wearable sensors to enable passive tracking of physiological responses (Morris and Aguilera 2012). This can provide researchers and clinicians with a more contextualised understanding of patients’ emotional states and begin to understand whether certain mindfulness practices are more significantly correlated to treatment outcomes. There are risks and limitations to involving technology in these processes including confidentiality and privacy and the possibility that lack of access to advanced technologies among low income, rural or elderly populations may increase disparities in mental health (Morris and Aguilera 2012). Given the significant role of mobile, social and wearable computing in people’s lives, future MBI research needs to be aware of developments and incorporate ways to make use of these technologies.

In summary, mindfulness research is at an early stage in the exploration of efficacy and effectiveness of MBIs. The literature identified in this review on home-practice and its relationship to clinical outcomes remains too scarce to speculate whether there is support for the benefits of home-practice as recommended by MBIs. Given the extensive time commitment required of participants to complete home-practice, it is critical to evaluate both experimentally and qualitatively the relationship of this practice and whether it improves clinical outcomes. In addition, the findings of this review illustrate the heterogeneity in the measurement of home-practice across studies. It is vital that the mindfulness research literature develop standardised and reliable measures to determine quantity and quality of home-practice that can be compared across studies. These developments would allow the mindfulness literature to determine more definitively the role of home-practice in MBIs and advance the literature on the mechanisms of intervention and process.

### Mindfulness Home-Practice Monitoring Form

Please complete the following record in between sessions, each time you practice. Also, make a note of anything that comes up during practice or any barriers to practice, so that we can talk about it at the next session. If you are not/no longer meeting with a therapist, please feel free to copy this form and use it for your own records.
